# COVID-19 infection prevention and control for hospital workers in Indonesia

**DOI:** 10.3389/fpubh.2023.1276898

**Published:** 2024-01-08

**Authors:** Robiana Modjo, Fatma Lestari, Hendra Tanjung, Abdul Kadir, Riskiyana Sukandhi Putra, Meilisa Rahmadani, Ali Syahrul Chaeruman, Fetrina Lestari, Juliana Sutanto

**Affiliations:** ^1^Occupational Health and Safety Department, Faculty of Public Health, Universitas Indonesia, Depok, West Java, Indonesia; ^2^Indonesia Occupational Health Experts Association, Jakarta, Indonesia; ^3^Disaster Risk Reduction Center, Universitas Indonesia, Depok, West Java, Indonesia; ^4^Directorate of Occupational Health and Sports, Ministry of Health, Jakarta, Indonesia; ^5^Universitas Indonesia Hospital, Depok, West Java, Indonesia; ^6^Department of Human Centred Computing, Monash University, Melbourne, VIC, Australia

**Keywords:** COVID-19 infection prevention and control, implementation of OHS in hospitals, occupational health, protection of hospital workers, Indonesian Ministry of Health Regulations

## Abstract

**Introduction:**

The outbreak of SARS-CoV-2 in 2019 led to a global pandemic, posing unprecedented challenges to healthcare systems, particularly in hospitals.

**Purpose:**

This study explores the intricacies of strategies employed for preventing and controlling COVID-19 in Indonesian hospitals, with a particular focus on the protocols, challenges, and solutions faced by healthcare professionals.

**Methods:**

Using a cross-sectional analysis, we examined 27 hospitals and uncovered disparities in their preparedness levels. During our investigation, we observed the robust implementation of infection prevention measures, which encompassed stringent protocols, adequate ventilation, and proper use of personal protective equipment. However, shortcomings were identified in areas such as surveillance, mental health support, and patient management.

**Discussion:**

This study underscores the importance of addressing these gaps, suggesting tailored interventions, and continuous training for healthcare staff. Effective leadership, positive team dynamics, and adherence to comprehensive policies emerge as pivotal factors. Hospitals should strengthen weak areas, ensure the ethical execution of emergency protocols, and integrate technology for tracking and improving standard operating procedures. By enhancing the knowledge and skills of healthcare workers and maintaining strong management practices, hospitals can optimize their efforts in COVID-19 prevention and control, thereby safeguarding the wellbeing of professionals, patients, and communities.

## 1 Introduction

In December 2019, a novel coronavirus, known as SARS-CoV-2, surfaced in Wuhan, China, giving rise to a global pandemic recognized as COVID-19 (1, 2). The swift transmission of the virus posed an unprecedented challenge to healthcare systems worldwide, requiring immediate and effective responses to mitigate its consequences (3). Hospitals, situated at the forefront of the battle against COVID-19, encountered significant challenges in ensuring the safety of healthcare professionals who were tirelessly working to treat and care for patients.

One of the risks healthcare professionals faced was their susceptibility to infection due to prolonged direct exposure to COVID-19 patients or contact with virus-contaminated surfaces, potentially leading to subsequent contact with their facial areas, including the eyes, nose, and mouth. Furthermore, specific healthcare procedures, such as endotracheal intubation and noninvasive ventilation, had the potential to aerosolize the virus, making it an airborne pathogen. This circumstance significantly increased the risk encountered by healthcare professionals (4–6).

Ensuring the safety of healthcare workers is of utmost importance not only for their wellbeing but also for the welfare of patients, staff, and the community they serve. Furthermore, it is essential for maintaining the effectiveness of the healthcare system during a pandemic (3, 7, 8). Therefore, prioritizing health and safety in hospitals is crucial for providing high-quality patient care, supporting the wellbeing of health workers, complying with the regulations, optimizing resources, and preparing for emergency situations. Nevertheless, implementing safety and health in hospitals during the COVID-19 pandemic was challenging. Numerous challenges were encountered, including insufficient resources (9, 10), overwhelmed healthcare systems (11), and healthcare staff experiencing fatigue and burnout (12).

Indonesia, like many other countries, grappled with the immense burden posed by the pandemic. Tragically, during the COVID-19 pandemic, a total of 2,087 healthcare professionals lost their lives, including 751 doctors, 670 nurses, 398 midwives, and 46 dentists, underscoring the significant impact of the virus on the country’s medical community (13). In addition, a crucial aspect of Indonesia’s fight against COVID-19 is how infection prevention and control measures are implemented in hospitals to safeguard the health and wellbeing of healthcare workers. Interestingly, Indonesia, as a diverse archipelagic nation with a large population, faced distinctive challenges in managing the transmission of the virus within healthcare facilities (14, 15).

Therefore, this study examines the strategies, protocols, and experiences of hospital workers in Indonesia as they grappled with the complexities of managing COVID-19 infections while continuing to provide essential healthcare services. Understanding the challenges faced by hospital workers in Indonesia, as well as the innovative solutions and best practices developed in response, is essential for shaping future policies and practices. This study delves into the multifaceted aspects of infection prevention and control in Indonesian hospitals, examining leadership aspects, coordination and communication, surveillance, risk communication, human resources, training for healthcare workers, and infection prevention and control.

## 2 Materials and methods

### 2.1 Research design and primary healthcare selection

**Figure 1:**
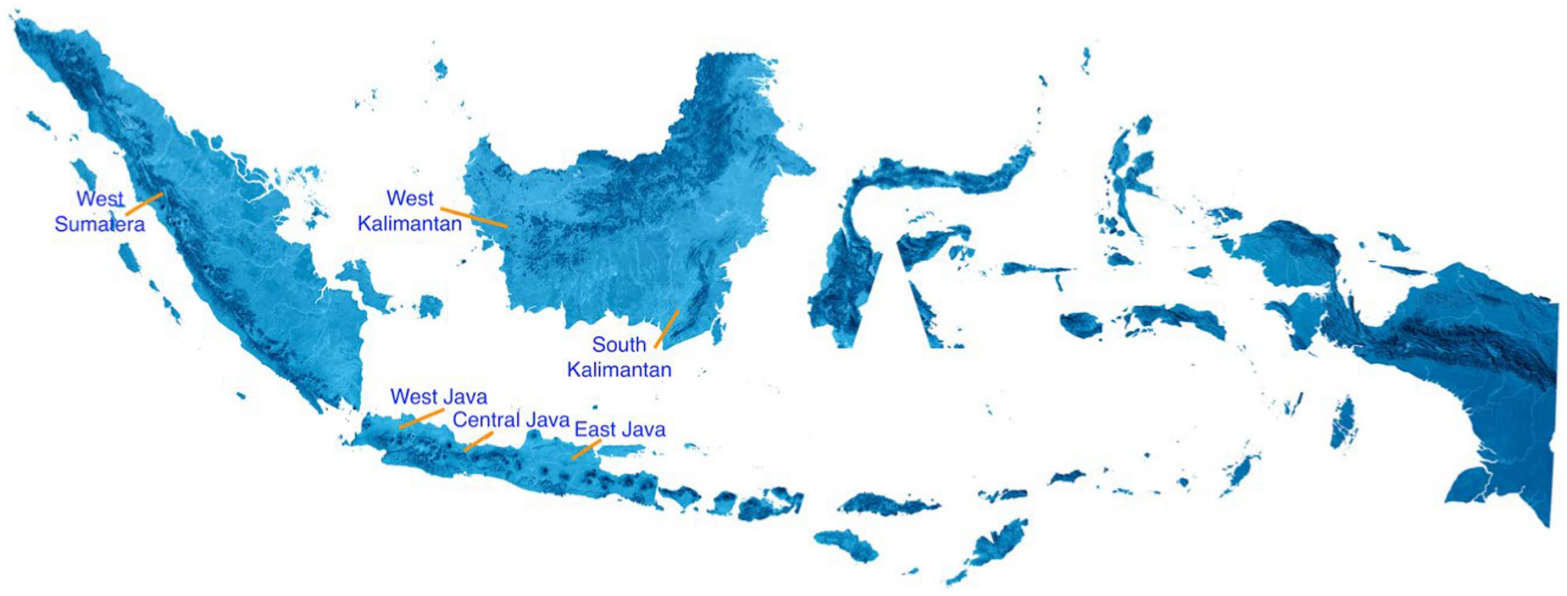
Locations of the selected 27 hospitals in six Indonesian provinces.

The research was conducted using a cross-sectional approach over a span of 6 months, from July to December 2020. The assessment of hospital workers’ protection was based on Ministry of Health Regulation No. 412 of the year 2020, which outlines guidelines for COVID-19 prevention and control, and Ministry of Health Regulation No. 327, which designates COVID-19 as a work-related disease for specific workers (7, 8). A total of 27 hospitals were selected from six provinces in Indonesia: West Java, Central Java, South Kalimantan, East Java, West Kalimantan, and West Sumatera. The distribution of hospitals across these provinces is as follows: West Java (4 hospitals), East Java (4 hospitals), Central Java (4 hospitals), South Kalimantan (4 hospitals), West Kalimantan (5 hospitals), and West Sumatera (6 hospitals). Figure 1 shows the locations of the selected hospitals in these six Indonesian provinces.

A non-probabilistic purposive sampling method was used in this study. The selected hospitals were all members of the network of the Indonesian Occupational Health Association (IOHA).

### 2.2 Research design and hospital selection

The selected hospitals were requested to complete a self-assessment evaluation form, which was distributed through the network of IOHA. These forms were filled out by key informants within the hospitals and then validated and verified through the local network of IOHA. The evaluation consisted of 12 elements, as detailed in Table 1. The scoring system was based on the degree of compliance with Ministry of Health Regulation No. 412 of the year 2020, which outlines guidelines for COVID-19 prevention and control, and Ministry of Health Regulation No. 327, which designates COVID-19 as a work-related disease for specific workers (16, 17). Compliance with these regulations was classified as “Very Good” if it exceeded 80%, “Good” if it ranged from 61 to 80%, “Sufficient” if it was between 41 and 60%, and “Poor” if it was ≤40%. The self-assessment evaluation form is available in Supplementary material.

**Table 1:** Evaluation elements and indicator criteria.

No.	Elements	Indicator criteria
0	General information	General information about the hospitals: type or hospital classification, number of beds for COVID-19 patients and non-COVID-19 patients, and number of workers
1	Leadership and incident management system	Good leadership and incident management system
2	Coordination and communication	Accurate and on-time coordination and communication to ensure risk analysis was informed for decision-making, effective collaboration, teamwork, and trust among all hospital workers and stakeholders; internal and external communication, and coordination with local and national authorities, including communities and primary healthcare
3	Surveillance and information management	Global surveillance and information management in hospitals
4	Risk communication and community participation	Risk communication and community participation to reduce pandemic hoax
5	Administration, finance, and business continuity	Administration, finance, and integral support to prevent, prepare, and respond to the pandemic
6	Human resources	Human resources to prevent, prepare, respond, and recover from the pandemic; it includes the requirements of hospital staff, procedures to ensure the capacity of the workers, and workers’ competencies to ensure the best quality services
7	Surge capacity	The ability of the hospital to manage a sudden request and progressive increase of COVID-19 cases, the ability of the hospital to provide care to COVID-19 patients with severe symptoms, and the ability to provide an adequate number of hospital staff
8	Sustainability of essential services	COVID-19 case management, hospitals’ business continuity, and providing essential services, including logistics and drugs
9	Patient management	Triage, patient referral, diagnosis, treatment, patient flow and tracing, post-hospital care follow-up, support services, logistics, supply chain, and pharmacies
10	Occupational health, mental health, and psychosocial support	Occupational health of the hospital workers, workers’ health promotion, mental health, and psychosocial support
11	Rapid identification and diagnosis	Rapid identification and diagnosis of COVID-19 to ensure effective identification of pandemic cases and rapid diagnosis support and services
12	Infection prevention and control	Procedures of the hospital for COVID-19 infection prevention and control

### 2.3 Data analysis, weighting, and score calculation

Data were collected and analyzed using an Excel sheet. The analysis involved univariate analysis, which entailed presenting important information related to variables and assessing compliance with Indonesia’s Ministry of Health regulations. Data were then visualized through the use of bar charts, spider diagrams, and compliance-level presentations.

### 2.4 Ethical considerations

The study was reviewed and approved by the ethics committee of the Research and Community Engagement of the Faculty of Public Health, Universitas Indonesia, under Ethics Approval Letter No. Ket-435/UN2.F10.D11/PPM.00.02/2020. Informed consent procedures were explained in detail during the ethics approval process and obtained consent prior to the commencement of the study. All methods applied for this study were performed in compliance with the relevant guidelines and regulations of the ethical committee. It is important to note that no animals or human subjects were used or involved in this study.

## 3 Results

### 3.1 Hospital information

The present study involves an analysis of diverse hospital parameters, including their class distinctions, bed capacity, ancillary amenities, and workforce composition, with a focus on their susceptibility to COVID-19. Furthermore, the assessment of COVID-19 management and control measures was conducted using a sample of hospitals categorized as Type A, Type B, Type C, and Type D. An examination of the acquired data reveals that Type A hospitals exhibit significantly superior metrics in terms of bed capacity, supporting facilities, and human resources compared to their counterparts in Type B, Type C, and Type D hospitals (Table 2).

**Table 2:** Hospitals’ general information.

No.	Hospital type	Acronyms of hospitals	Provinces	Number of beds for COVID-19 patients	Number of hospital beds for routine care	Number of workers
1	Type A	RS DSS	East Java	1,609	351	4,976
2		RS HSB	West Java	288	131	3,867
3		RS DMD	West Sumatera	800	186	2,814
4		RS MWS	Central Java	906	195	2,316
5		RS ULB	South Kalimantan	656	160	2,138
6	Type B	RS KJB	East Java	524	84	1,452
7		RS CBC	West Java	249	39	1,043
8		RS DSD	South Kalimantan	112	89	927
9		RS MNS	West Sumatra	275	17	890
10		RS SLT	Central Java	267	28	857
11		RS MDL	East Java	216	47	784
12		RS AAZ	West Kalimantan	257	11	751
13		RS PMK	East Java	258	31	671
14		RS RZM	South Kalimantan	130	16	310
15	Type C	RS IKB	South Kalimantan	307	48	999
16		RS CDM	West Java	285	8	746
17		RS SMP	West Sumatera	144	95	508
18		RS PBC	West Java	118	7	416
19		RS PDP	West Sumatera	150	32	450
20		RS PMW	Central Java	104	12	404
21		RS SJJ	West Sumatera	109	8	376
22		RS UAP	West Sumatera	139	71	364
23		RS KTH	West Kalimantan	138	6	253
24		RS SRT	Central Java	105	15	220
25		RS PTJ	South Kalimantan	6	37	203
26		RS MDS	Kalimantan Barat	207	71	48
27	Type D	RS PTK	West Kalimantan	50	12	101

### 3.2 COVID-19 confirmed cases in hospital workers

Table 3 presents a comprehensive overview of COVID-19 incidence among the workforce in the 27 hospitals under scrutiny. MWS Hospital reported the highest number of positive cases, with 219 out of 2,316 total workers tested positive. Remarkably, three hospitals, namely, RS PBC, SJJ Hospital, and PTK Hospital, reported no instances of COVID-19 among their employees. Moreover, 11 hospitals had an equal number of workers who tested positive for COVID-19 and those who had recovered. This suggests that no fatalities occurred within these hospitals, and all affected workers had successfully recuperated by the time of data collection. Conversely, six hospitals documented cases of worker mortality due to COVID-19. Notably, DSS Hospital reported the highest fatality rate, with 10 individuals, including 4 healthcare workers and 6 non-healthcare workers, succumbing to the virus.

**Table 3:** Confirmed cases of COVID-19 in hospital workers.

No.	Hospital type	Acronyms of hospitals	Province	Number of workers	Number of workers who tested positive for COVID-19		Number of workers who recovered from COVID-19		Number of workers who died from COVID-19
					Healthcare workers	Non-healthcare workers	Healthcare workers	Non-healthcare workers	
1	Type A	RS MWS	Central Java	2,316	195	24	195	25	1
2		RS DMD	West Sumatera	2,814	155	49	155	49	0
3		RS ULB	South Kalimantan	2,138	141	52	139	50	2
4		RS DSS	East Java	4,976	84	15	80	9	10
5		RS HSB	West Java	3,867	57	4	54	4	0
6	Type B	RS RZM	South Kalimantan	310	53	18	53	18	0
7		RS MNS	West Sumatera	890	23	3	4	0	0
8		RS PMK	East Java	671	16	2	15	2	1
9		RS CBC	West Java	1,043	7	10	7	10	0
10		RS MDL	East Java	784	14	3	14	3	0
11		RS KJB	East Java	1,452	12	5	12	4	0
12		RS SLT	Central Java	857	2	0	2	0	0
13		RS AAZ	West Kalimantan	751	2	0	1	0	0
14		RS DSD	West Kalimantan	927	0	1	0	0	1
15	Type C	RS IKB	South Kalimantan	999	65	13	58	12	0
16		RS SMP	West Sumatera	508	33	2	28	2	0
17		RS PTJ	South Kalimantan	203	22	5	22	5	0
18		RS PDP	West Sumatera	450	21	3	21	3	0
19		RS UAP	West Sumatera	364	15	6	13	4	1
20		RS PMW	Central Java	404	9	0	9	0	0
21		RS KTH	West Kalimantan	253	8	1	8	1	0
22		RS CDM	West Java	746	5	2	5	1	0
23		RS SRT	Central Java	220	2	0	2	0	0
24		RS MDS	West Kalimantan	48	0	1	0	1	0
25		RS PBC	West Java	416	-	-	-	-	0
26		RS SJJ	West Sumatera	376	-	-	-	-	0
27	Type D	PTK	West Kalimantan	101	-	-	-	-	0

### 3.3 Compliance with COVID-19 prevention and control regulations

**Figure 2:**
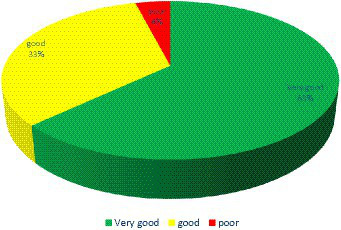
Compliance with regulations for COVID-19 prevention and control in hospitals.

Table 4 presents the compliance percentages of various hospitals with the COVID-19 prevention and control guidelines established by the Indonesian Ministry of Health. RS RST exhibited the lowest compliance rate at 52.97%, while RS PTJ demonstrated the highest adherence, with a score of 98.51%. The data analysis revealed that 63% of the sampled hospitals achieved very good implementation, with a score exceeding 80%. Additionally, 33% of the hospitals were classified as having good implementation, with scores ranging from 60 to 80%. Conversely, a mere 4% of the hospitals demonstrated inadequate implementation, scoring below 60%, as illustrated in Figure 2.

**Table 4:** Compliance percentages of hospitals with COVID-19 prevention and control guidelines.

No.	Acronyms of hospitals	Total score	Compliance (%)
1	RS KJB	950	94.06
2	RS PMK	625	61.88
3	RS MDL	970	96.04
4	RS DSS	970	96.04
5	RS SRT	535	52.97
6	RS PMW	825	81.68
7	RS SLT	795	78.71
8	RS MWS	940	93.07
9	RS DSD	815	80.69
10	RS AAZ	945	93.56
11	RS MDS	885	87.62
12	RS PTK	715	70.79
13	RS KTH	840	83.17
14	RS ULB	875	86.63
15	RS RZM	660	65.35
16	RS IKB	930	92.08
17	RS PTJ	995	98.51
18	RS DMD	950	94.06
19	RS SMP	840	83.17
20	RS UAP	865	85.64
21	RS MNS	715	70.79
22	RS SJJ	645	63.86
23	RS PDP	815	80.69
24	RS HSB	920	91.09
25	RS CBC	730	72.28
26	RS CDM	765	75.74
27	RS PBC	705	69.80

**Figure 3:**
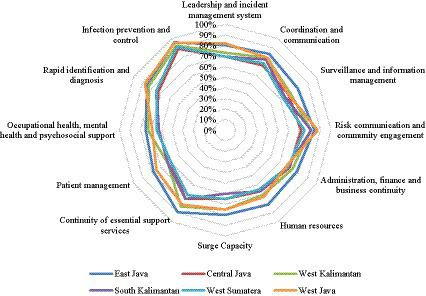
Evaluation of hospital COVID-19 preparedness in six provinces.

Upon closer examination of each element of regulation within the areas of study, the data reveal that infection prevention and control exhibited the highest level of compliance, standing at an impressive 92%. Following closely behind are risk communication and community engagement and rapid identification and diagnosis, all at 80%. The lowest levels of compliance were observed in the areas of surveillance and information management; occupational health, mental health, and psychosocial support; and patient management, with scores of 69, 68, and 66%, respectively. When considering different geographic areas, East Java emerged as the leader, with a mean score of 83%. West Kalimantan secured the second position with a score of 77%, while South Kalimantan, West Java, and Central Java achieved scores of 71, 70, and 70%, respectively (Table 5). Figure 3 shows a radar plot analysis, providing an overview of COVID-19 prevention and control assessments in hospitals across the six areas. Strong performance is evident in the elements of infection prevention and control and risk communication and community engagement. In addition, the evaluation of COVID-19 preparedness for each element within the 27 hospitals is displayed, with red areas indicating a score below 60%, categorized as poor implementation Attachment S1.

**Table 5:** Mean score evaluation for each element and area of COVID-19 prevention and control in hospitals.

No.	Evaluation elements	East Java	Central Java	West Kalimantan	South Kalimantan	West Kalimantan	West Java	Average
1	Leadership and incident management system	80%	70%	73%	70%	70%	82%	74%
2	Coordination and communication	83%	71%	79%	77%	73%	79%	77%
3	Surveillance and information management	79%	69%	71%	65%	63%	69%	69%
4	Risk communication and community engagement	84%	72%	81%	81%	75%	88%	80%
5	Administration, finance, and business continuity	78%	70%	73%	69%	69%	67%	71%
6	Human resources	81%	67%	71%	67%	65%	73%	70%
7	Surge capacity	80%	65%	75%	60%	65%	75%	70%
8	Continuity of essential support services	90%	75%	83%	75%	71%	81%	79%
9	Patient management	78%	59%	63%	63%	59%	75%	66%
10	Occupational health, mental health, and psychosocial support	75%	65%	73%	63%	65%	69%	68%
11	Rapid identification and diagnosis	85%	73%	83%	75%	75%	88%	80%
12	Infection prevention and control	96%	89%	92%	91%	91%	96%	92%
	Mean	83%	70%	77%	71%	70%	70%	78%

## 4 Discussion

The findings of this study have brought to light significant disparities in the preparedness and response capabilities of hospitals in the face of the COVID-19 pandemic. The World Health Organization (WHO) emphasized the escalating demand for healthcare systems, a direct consequence of the rising number of COVID-19 cases. This surge has particularly underscored the criticality of augmenting hospital bed capacities to cater to the exigencies of the most severe cases within the prevailing pandemic context (18). Addressing this challenge necessitates the implementation of a range of interventions aimed at curtailing and mitigating the virus’s transmission within healthcare facilities (19). In addition, the comprehensive analysis of various hospital parameters was crucial according to the implementation of COVID-19 prevention and control for hospital workers in Indonesia by referring to Ministry of Health Regulation Number 413 of 2020 and Indonesian Ministry of Health Regulation No. 327 of the year 2020, which provided valuable insights into the susceptibility of different hospitals to COVID-19. Our study categorized hospitals into four types—A, B, C, and D—based on the following parameters: leadership and incident management system; coordination and communication; surveillance and information management; risk communication and community engagement; administration, finance, and business continuity; human resources; surge capacity; sustainability of essential services; patient management; occupational health, mental health, and psychosocial support; rapid identification and diagnosis; and infection prevention and control.

Our study assessed a hospital’s compliance with COVID-19 regulations across various elements. Strong implementation was seen in infection prevention and control, rapid identification and diagnosis, and risk communication. However, areas needing improvement included surveillance and information management, occupational health, mental health, psychosocial support, and patient management.

In infection prevention, most hospitals ensure adequate PPE, ventilation, and hygiene measures. Moreover, hospitals effectively controlled the spread of COVID-19 through strategic measures. Occupational Health and Safety Officers ensured PPE availability, coordinating with units and monitoring usage. Given the airborne transmission risk during medical procedures and treatments, stringent precautions were advised, mandating the utilization of PPE, such as masks, coveralls, gloves, and goggles (20). Furthermore, standard operational procedures (SOPs) for prevention, control, and management were rigorously maintained and updated. Isolation areas with proper ventilation and clear instructions were established. The implementation of SOPs for COVID-19 prevention, control, and management was rigorously maintained. These SOPs, which are vital in preventing COVID-19 dissemination, were subject to periodic reviews and updates, accommodating the evolving nature of the pandemic (21). Hand hygiene stations were strategically placed, and multilingual educational posters were used. Staff were trained per WHO guidelines, emphasizing hand hygiene, social distancing, and proper PPE use. Beds were spaced 1 m apart regardless of COVID-19 status, ensuring infection prevention. Crucially, infection prevention and control were bolstered by the strategic allocation of isolation areas equipped with clear instructions, adequate supplies, and effective ventilation systems. Utilizing negative-pressure rooms with controlled airflow, especially when using mechanical ventilation, was imperative to prevent cross-contamination. Ventilation systems, maintaining optimal thermal conditions and adequate airflow, were acknowledged as fundamental in healthcare facilities by authoritative bodies, such as the Pan American Health Organization (PAHO). A ventilation rate of 6 to 12 air changes per hour, with 12 ACH being ideal for new constructions, was recommended (22).

In addition to these technical measures, the hospitals emphasized general preventive strategies. Entry and hospital areas were equipped with essential facilities for hand hygiene, including water, soap, tissue, hand sanitizers, and strategically placed trash cans. Encouragingly, the populace was educated on appropriate handwashing practices and advised to maintain social distancing (23). Hospitals disseminated information through multilingual posters, ensuring clarity and understanding and covering topics such as hand hygiene, respiratory etiquette, and social distancing. Furthermore, hospital staff underwent rigorous training in adherence to Regulation 413/WHO guidelines, focusing on meticulous hand hygiene, respiratory etiquette, social distancing, and the proper utilization of personal protective equipment. Policies mandating a minimum 1-m spacing between beds, irrespective of suspected COVID-19 cases, were established and diligently enforced.

Rapid identification involved staff training, communication systems, and sample handling. Risk communication protocols were effectively disseminated to staff, patients, and the community. Our study found that key indicators in rapid COVID-19 identification emphasized intensive staff training. Prompt diagnosis and reporting were crucial due to the virus’s novelty, necessitating swift screening to identify clinical symptoms (24). The hospital ensured comprehensive training, focusing on hygiene and workplace controls during outbreaks (25). A robust communication system covering entrances and patient areas facilitated rapid case reporting. Informative materials in labs aided safe sample handling and disseminated vital health information (26). In addition, emergency departments implemented efficient triage procedures, promptly isolating and assessing patients with respiratory symptoms. This comprehensive approach underscored the hospital’s commitment to efficient and accurate COVID-19 diagnosis and management. Furthermore, in a serious pandemic situation, there has been a shift in messaging, emphasizing the importance of supporting precautions when communicating about the crisis. Given these circumstances, it is crucial to assist organizations and institutions in achieving their goals and missions. Encouraging public trust and preventing harm and disruption are essential (27). Additionally, to ensure primary healthcare and promote people-centered services, it is crucial to consider community engagement within the healthcare sector. Involvement and participation among communities are viewed as critical in various sectors, including healthcare facilities. During the COVID-19 pandemic, the resilience of communities became a vital component in building trust and minimizing the spread of the virus (28, 29).

Surveillance and information management lacked in data reporting and integration. Hospital workers, both healthcare and non-healthcare workers, have been informed and trained on the definition of COVID-19 cases, including the definition of close contact and quarantine systems. Standard forms for reporting COVID-19 case information to a centralized health information system (Team/Task Force/Committee) within 24 h of identifying cases are available. SOPs for collecting, confirming, and validating COVID-19 data for designated workers are also in place. However, hospitals with low scores indicate a lack of several indicators in this element. According to Morgan et al. (30), the uncontrolled spread of the virus was due to the weak surveillance of cases and insufficient capacity of institutions or countries to integrate data for public health controls. It was also reported that some countries had poor data management and dissemination of data to residents. Furthermore, addressing infection prevention could be achieved by utilizing information technology in each phase of the crisis, such as a pandemic, especially for public health surveillance. Information technology has been employed to enhance the speed of diagnosis, improve epidemic management, monitor the situation, and deliver important information related to health promotion (31, 32).

Another concern in prevention and control programs in health settings is psychosocial issues. It was seen that occupational health and mental health support needed attention due to high stress levels among healthcare workers. It identified the profound issue of psychosocial challenges during pandemic situations. The research found a high prevalence of mental health problems among healthcare employees, particularly those dealing with COVID-19 patients. These problems encompass not only anxiety, insomnia, and depression but also somatization and post-traumatic stress symptoms (33). A study conducted in Toronto revealed alarming statistics: 30.4% of health professionals experienced burnout syndrome, 44.9% suffered from psychosocial distress, and 13.8% exhibited post-traumatic stress symptoms (34, 35). Visheh et al. (36) emphasized that healthcare workers face immense challenges due to highly infectious diseases. They often worked in isolated areas without adequate education and training, leading to a decline in their mental health. Consequently, it is crucial to implement appropriate measures to address these issues, including supportive interventions, encouragement, motivation, protective measures, and educational and training interventions.

Several fundamental indicators for preventing and controlling COVID-19 in hospitals include occupational health, mental health, and psychosocial support, including providing protection, training, and personal protective equipment (PPE) for hospital workers offering medical services to suspected, probable, or confirmed COVID-19 cases. This includes screening, resuscitation, initial stabilization, supportive therapy, and prevention of complications. A risk management process should be implemented in hospitals to prevent and control COVID-19 in every work area or location related to potential worker exposure. Policies and capacities for managing occupational safety and health, coupled with infection prevention and control measures, should be in place to safeguard hospital staff, suspected cases, their families, and close contacts.

The patient management aspect was the weakest element implemented in the study hospitals. Indicators in this category include hospitals updating protocols for essential care services for COVID-19 patients based on WHO guidelines, Ministry of Health regulations, regional policies, and hospital protocols. In addition, it is recommended to establish functioning procedures for receiving and transferring patients to isolation areas within the hospital, along with providing the necessary therapeutic and diagnostic support services.

Current best practices, such as placing all suspected patients in droplet masks during assessments and transit, restricting visitors, and implementing appropriate standard precautions, have been suggested for isolating and controlling infections in confirmed and suspected cases, as noted by Jamil et al. (37). Healthcare professionals should be familiar with these procedures to avoid mistakes (38). The WHO emphasizes that infection prevention and control are as critical as clinical patient management, healthcare professional safety, and preventing hospital-acquired infections. Therefore, implementing administrative, environmental, and engineering controls is crucial in healthcare facilities (39). Furthermore, healthcare facilities need to establish protocols for untested, trial, or emergency measures that have not been clinically approved, as well as ethically approved clinical trials or the Monitored Emergency Use of Unregistered Interventions (MEURI framework), all of which should be closely monitored. Hospitals must also ensure the implementation of infection prevention and control protocols, establish a safe hospital or health facility network, and provide transportation services for patient referrals, including transfers from home care services, if available.

Leadership and incident management systems were crucial, demanding effective policies and positive leader–team relationships to navigate the challenges posed by the pandemic. Effective leadership and incident management systems are vital for the success of COVID-19 prevention and control efforts (40). Rapid responses have been crucial in understanding and addressing COVID-19. The implementation of policies to combat COVID-19 has directly impacted institutional leadership, making them instrumental in guiding organizational teams. Therefore, fostering positive relationships between leaders and teams is essential (41). Positive social interactions in the workplace can nurture trust, respect, and support, enhancing employee performance (42). Furthermore, the COVID-19 pandemic poses challenges for leaders, testing their ability to handle cases and implement efficient incident management systems (IMSs) (43). It is crucial to design an IMS tailored for each country to bolster follow-up and support during crises, including pandemics (44).

It is important to acknowledge the strengths and limitations of this study. The in-depth analysis of hospital factors and adherence to regulations offers valuable insights into the pandemic-related challenges hospitals encounter. Nonetheless, it is essential to note that the study’s scope is limited to a particular area and might not apply universally. Moreover, relying on historical data may not reflect current, real-time changes in hospital readiness and responses. Future studies could benefit from additional analyses employing advanced methods. However, important implications and areas for further exploration according to our study are improving hospital preparedness through policy implementation by encouraging hospitals and healthcare systems to implement and adhere to comprehensive policies, particularly focusing on weak areas identified in our study, such as surveillance and information management, mental health support, and patient management protocols. In addition, enhancing skills related to rapid identification, information management, and patient care, especially for mental health support, are also crucial to be provided by healthcare institutions. To support the psychosocial aspects, designing interventions that address the mental health and psychosocial challenges faced by healthcare workers also needs to be considered. In terms of prevention and control, prevention programs should be implemented that integrate technology for better tracking of protocols and PPE usage, emphasize continuous improvement, regularly update SOPs, and ensure ethical considerations in emergency measures and protocols. Improving leadership and incident management and promoting positive team relationships for effective collaboration is vital for addressing the challenges presented by the pandemic.

## 5 Conclusion

This study examines the current state of COVID-19 prevention and control implementation in healthcare settings. In conclusion, the study reveals that the majority of hospitals achieved good scores, indicating full compliance with the regulations that minimize the spread of COVID-19 in the workplace and mitigate its impact on various aspects. The three elements that received the highest scores are infection prevention and control, risk communication and communication engagement, and rapid identification and diagnosis. However, the areas with the most room for improvement are surveillance and information management; occupational health, mental health, and psychosocial support; and patient management. Addressing these aspects necessitates a strong commitment to hospital management, ultimately safeguarding the health and wellbeing of healthcare professionals, patients, and communities from the threat of COVID-19. Providing healthcare workers with updated and regular training is vital to ensure their proficiency and procedures when dealing with individuals affected by COVID-19. This effort has yielded positive impacts, but it is important for hospital management to maintain focus on areas that received moderate scores, such as human resources; surge capacity; administration, finance, and business continuity; coordination and communication; and leadership and incident management systems. By addressing these areas, hospitals can attain the highest level of COVID-19 prevention and control implementation in the workplace.

## Data availability statement

The original contributions presented in the study are included in the article/Supplementary material, further inquiries can be directed to the corresponding author.

## Author contributions

RM: Conceptualization, Methodology, Resources, Validation, Visualization Writing – original draft, Writing – review & editing, Data curation, Formal analysis, Funding acquisition, Investigation, Supervision. FaL: Conceptualization, Data curation, Investigation, Methodology, Supervision, Validation, Writing – original draft, Writing – review & editing. HT: Conceptualization, Data curation, Investigation, Resources, Software, Validation, Writing – original draft, Writing – review & editing. AK: Conceptualization, Methodology, Resources, Software, Validation, Visualization, Writing – original draft, Writing – review & editing. RP: Investigation, Resources, Supervision, Validation, Visualization, Writing – original draft, Writing – review & editing. MR: Investigation, Methodology, Project administration, Validation, Visualization, Writing – original draft, Writing – review & editing. AC: Investigation, Methodology, Project administration, Validation, Visualization, Writing – original draft, Writing – review & editing. FeL: Investigation, Methodology, Project administration, Resources, Visualization, Writing – original draft, Writing – review & editing. JS: Conceptualization, Methodology, Software, Validation, Visualization, Writing – review & editing.
