# Supplementary material for: COVID-19 infection prevention and control for hospital workers in Indonesia
Source: Front Public Health. 2024 Jan 8;11:1276898. doi: 10.3389/fpubh.2023.1276898 (PMC10800904; doi:10.3389/fpubh.2023.1276898)
Supplement: Supplementary file 1 [file Table_1.docx]

Attachment 1: Evaluation of COVID-19 preparedness for each element in 27 hospitals

| **No** | **Evaluation element** | **RS KJB** | **RS PMK** | **RS MDL** | **RS DSS** | **RS SRT** | **RS PMW** | **RS SLT** | **RS MWS** | **RS DSD** | **RS AAZ** | **RS MDS** | **RS PTK** | **RS KTH** | **RS ULB** | **RS RZM** | **RS IKB** | **RS PTJ** | **RS DMD** | **RS SMP** | **RS UAP** | **RS MNS** | **RS SJJ** | **RS PDP** | **RS HSB** | **RS CBC** | **RS CDM** | **RS PBC** |
| --- | --- | --- | --- | --- | --- | --- | --- | --- | --- | --- | --- | --- | --- | --- | --- | --- | --- | --- | --- | --- | --- | --- | --- | --- | --- | --- | --- | --- |
|  |  | **(%)** | **(%)** | **(%)** | **(%)** | **(%)** | **(%)** | **(%)** | **(%)** | **(%)** | **(%)** | **(%)** | **(%)** | **(%)** | **(%)** | **(%)** | **(%)** | **(%)** | **(%)** | **(%)** | **(%)** | **(%)** | **(%)** | **(%)** | **(%)** | **(%)** | **(%)** | **(%)** |
| 1 | Leadership and incident management system | 79 | 50 | 93 | 100 | 36 | 64 | 79 | 100 | 86 | 100 | 93 | 93 | 93 | 86 | 64 | 100 | 100 | 86 | 79 | 86 | 57 | 64 | 86 | 100 | 50 | 71 | 43 |
| 2 | Coordination and communication | 92 | 50 | 92 | 100 | 42 | 83 | 83 | 83 | 67 | 100 | 75 | 92 | 83 | 67 | 42 | 100 | 92 | 92 | 100 | 100 | 83 | 42 | 92 | 83 | 100 | 83 | 73 |
| 3 | Surveillance and information management | 92 | 58 | 83 | 83 | 50 | 67 | 58 | 75 | 75 | 100 | 67 | 75 | 100 | 83 | 50 | 100 | 100 | 100 | 83 | 83 | 67 | 58 | 58 | 75 | 58 | 83 | 67 |
| 4 | Risk communication and community engagement | 100 | 38 | 100 | 100 | 50 | 75 | 100 | 75 | 100 | 75 | 88 | 100 | 100 | 63 | 63 | 100 | 100 | 88 | 88 | 88 | 38 | 38 | 88 | 88 | 50 | 75 | 75 |
| 5 | Administration, finance, and business continuity | 75 | 50 | 100 | 88 | 44 | 63 | 81 | 88 | 38 | 88 | 81 | 63 | 69 | 75 | 44 | 81 | 100 | 100 | 69 | 69 | 56 | 44 | 63 | 81 | 56 | 75 | 44 |
| 6 | Human resources | 100 | 33 | 92 | 100 | 42 | 50 | 75 | 92 | 75 | 83 | 75 | 50 | 100 | 100 | 92 | 100 | 100 | 92 | 75 | 100 | 25 | 42 | 83 | 92 | 67 | 50 | 42 |
| 7 | Surge Capacity | 100 | 40 | 100 | 80 | 40 | 80 | 40 | 100 | 80 | 80 | 80 | 60 | 70 | 100 | 40 | 60 | 100 | 100 | 70 | 70 | 20 | 50 | 30 | 80 | 50 | 80 | 50 |
| 8 | Continuity of essential support services | 100 | 58 | 100 | 100 | 42 | 92 | 67 | 83 | 83 | 100 | 100 | 50 | 67 | 100 | 58 | 100 | 100 | 100 | 92 | 100 | 92 | 75 | 92 | 92 | 67 | 67 | 67 |
| 9 | Patient management | 100 | 38 | 75 | 100 | 25 | 50 | 75 | 88 | 88 | 75 | 63 | 63 | 63 | 100 | 25 | 75 | 100 | 63 | 63 | 63 | 75 | 63 | 63 | 100 | 88 | 63 | 63 |
| 10 | Occupational health, mental health, and psychosocial support | 75 | 42 | 100 | 83 | 33 | 75 | 58 | 92 | 50 | 83 | 83 | 33 | 58 | 50 | 50 | 50 | 100 | 83 | 42 | 33 | 42 | 42 | 58 | 67 | 83 | 33 | 25 |
| 11 | Rapid identification and diagnosis | 100 | 42 | 100 | 100 | 50 | 83 | 67 | 100 | 100 | 100 | 92 | 67 | 92 | 75 | 42 | 92 | 100 | 83 | 75 | 75 | 100 | 58 | 67 | 100 | 25 | 67 | 83 |
| 12 | Infection prevention and control | 100 | 86 | 98 | 100 | 71 | 100 | 93 | 100 | 89 | 98 | 96 | 70 | 82 | 95 | 88 | 100 | 96 | 100 | 98 | 96 | 89 | 86 | 96 | 98 | 88 | 88 | 95 |
